# High‐Pressure CO Electroreduction at Silver Produces Ethanol and Propanol

**DOI:** 10.1002/anie.202108902

**Published:** 2021-08-25

**Authors:** Stefan J. Raaijman, Maarten P. Schellekens, Paul J. Corbett, Marc T. M. Koper

**Affiliations:** ^1^ Leiden Institute of Chemistry Leiden University PO Box 9502 2300 RA Leiden The Netherlands; ^2^ Shell Technology Centre Amsterdam Shell Global Solutions International B.V. Grasweg 31 1031 HW Amsterdam The Netherlands

**Keywords:** alcohol, C−C coupling, CO reduction, liquid fuel, silver

## Abstract

Reducing CO_2_ to long‐chain carbon products is attractive considering such products are typically more valuable than shorter ones. However, the best electrocatalyst for making such products from CO_2_, copper, lacks selectivity. By studying alternate C_2+_ producing catalysts we can increase our mechanistic understanding, which is beneficial for improving catalyst performance. Therefore, we investigate CO reduction on silver, as density functional theory (DFT) results predict it to be good at forming ethanol. To address the current disagreement between DFT and experimental results (ethanol vs. no ethanol), we investigated CO reduction at higher surface coverage (by increasing pressure) to ascertain if desorption effects can explain the discrepancy. In terms of product trends, our results agree with the DFT‐proposed acetaldehyde‐like intermediate, yielding ethanol and propanol as C_2+_ products—making the CO_2_ electrochemistry of silver very similar to that of copper at sufficiently high coverage.

## Graphical abstract

Reducing CO_2_ to long‐chain carbon products is of great interest. Here, CO reduction was studied on silver at high surface coverage, as DFT results predict it to be good at forming ethanol. The results agree with the DFT‐proposed acetaldehyde‐like intermediate, yielding ethanol, ethylene glycol and propanol as C_2+_ products—making the CO_2_ electrochemistry of silver very similar to that of copper at sufficiently high coverage.

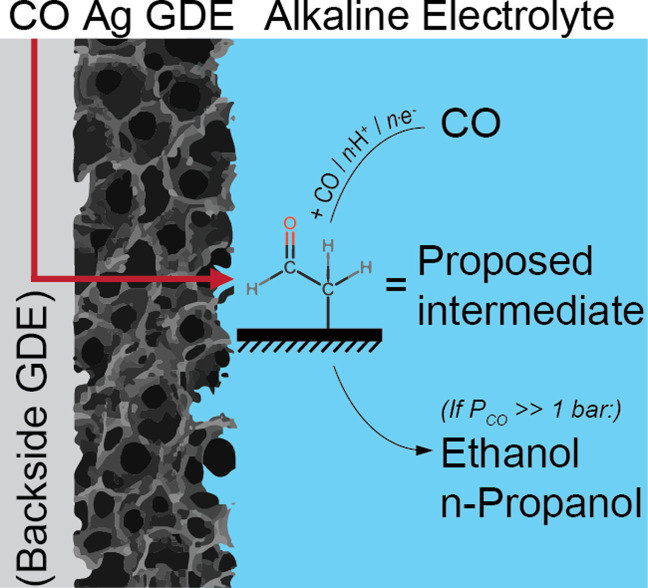

Few electrocatalytic systems are known to be capable of generating carbon‐coupled products from the CO_2_ reduction reaction (CO2RR) and/or the CO reduction reaction (CORR).^[1]^ Out of these, copper is by far the most capable electrocatalyst for making C_2+_ molecules, yielding ethylene,^[2]^ ethanol,^[3]^ and *n*‐propanol^[4]^ as its primary multi‐carbon products.[^1d^, ^5^] Other catalysts (in aqueous media) include molybdenum disulfides,^[6]^ enzymatic nitrogenases with a vanadium/molybdenum active center^[7]^ (and its organometallic homologues^[8]^), bimetallic palladium/gold nanparticles,^[9]^ heteroatom (N, B)‐doped nanoparticles,^[10]^ transition‐metal (Ni, Fe)‐doped carbon xerogels,^[11]^ certain surfaces when coated with functionalized films,^[12]^ nickel/gallium alloys,^[13]^ nickel phosphides,^[14]^ and metallic nickel and silver.^[15]^ However, these non‐copper catalysts exhibit comparatively low (on the order of a few %) faradaic efficiencies (FEs) for C_2+_ products.

As for the currently existing theories on the C−C coupling mechanism, an in‐depth review concerning non‐copper systems has recently been published by Zhou and Yeo,^[16]^ whilst comprehensive reviews regarding the mechanism on copper can be found, for example, here^[17]^ and in a review by Fan et al.^[18]^ who compare mechanisms on a per‐product basis. For comprehensibility, summaries of the main theories for making C_2_ and C_3_ products on metallic Cu in aqueous media are also provided in the Supporting Information (SI) in Schemes A‐C2 to I‐C2 (with, where applicable, reaction paths to C_3_ products in accompanying Schemes A‐C3 to J‐C3).

To increase molecular‐level understanding of the formation mechanism for C_2+_ products, Hanselman et al. carried out density functional theory (DFT) calculations on CO reduction to C_2_ products for various transition‐metal surfaces (including silver), suggesting two reaction pathways: one to ethylene and one to ethanol, bifurcating from a surface intermediate that is one hydrogen short of acetaldehyde.^[19]^ This mechanism, where acetaldehyde is the precursor to ethanol, agrees with experiments on copper single‐crystal electrodes.^[20]^ Their DFT calculations indicate that, among nine transition‐metal surfaces, only copper has a reasonably low onset potential for ethylene formation whilst ethanol has a slightly later onset. The former agrees well with literature as copper is reported to yield reasonable FE towards C_2_H_4_ at overpotentials of a few hundred mV,[^2a^, ^21^] although experimentally no large differences are observed between the formation onsets of ethylene and ethanol.[^2b^, ^22^] Importantly, their calculations also indicate silver should have a lower onset potential for ethanol formation than copper whilst being incapable of producing ethylene. In chemical terms, silver is seemingly too noble to break the last C−O bond.

This prediction is, however, in apparent disagreement with experimental studies as the maximum reported FE of CO_2_ to ethanol is ca. 0.1 % on silver vs. 40 % on copper.[^3^, ^15a^, ^22a^] Hanselman et al. hypothesized this disagreement may be a consequence of CO desorbing rather than reacting further on silver due to its unfavorable adsorption strength.^[19]^ Hence, herein we probe the validity of the theory that silver can produce ethanol if the CO coverage on the surface is sufficiently high. To this end, we study CO reduction at elevated pressure as a means of increasing surface coverage which enhances the likelihood of (intermolecular) reactions involving CO_ads_. In line with DFT calculations we observe ethanol (whose formation is positively influenced by increasing the pressure) and no ethylene during CORR. Furthermore, ethylene glycol and *n*‐propanol are also observed and found to exhibit a similar pressure dependency as ethanol, providing us with additional insight into carbon−carbon bond formation and the mechanistic aspects of C_3_ production.

Experiments were carried out in a three‐compartment electrochemical cell inside an autoclave that could be pressurized up to 60 barg, with the gaseous products leaving the cell analyzed by gas chromatography, and liquid products analyzed by NMR. The working electrode was a silver gas diffusion electrode (GDE) with a 1 cm^2^ exposed geometrical area. Alkaline conditions were employed as these promote C_2_ formation from CO on copper.^[23]^ A Ag|AgCl|KCl (3 m) reference was used as a reference electrode and potentials are reported on this scale unless denoted otherwise. Reported potentials are not IR‐corrected because of the inherent inhomogeneity of the interfacial potential on a GDE, rendering the nominal reported potentials unrepresentative of the “real” potential. As a figure of merit, the nominal IR‐corrected potential of the most negative potential employed in this work (−4.5 V) was calculated to be ca. −1 V vs. RHE (see SI). A comprehensive description of the experimental setup can be found in the SI, including control experiments conducted in the absence of CO and in the absence of applied potential in the presence of CO to prove that the products we report are indeed the result of electrochemical CO reduction.

Absolute formation rates of CORR‐related products obtained for CO reduction in 0.5 m KOH on a silver GDE at various potentials are depicted in Figure 1 a–c, for reactant (carbon monoxide) pressures ranging between 10 and 60 barg. Investigated reaction times were between 2.6 and 73 hours, with more positive potentials necessitating longer times to guarantee a minimum of charge had passed. The CORR products depicted in Figure 1 are minority species, with hydrogen and formate (Figure S3a and S3b, respectively) being the main products. As we study the carbon−carbon bond formation mechanism on silver, we will disregard H_2_ and HCOO^−^ as neither is the result of CO reduction or contains a C−C bond. However, to briefly address the possible origin of formate (being in equal oxidation state as CO), we refer the reader to literature wherein formate is proposed to form through a solution phase reaction between CO and hydroxide, which may occur in this work given the high electrolyte alkalinity and elevated carbon monoxide pressures.^[24]^

**Figure 1:**
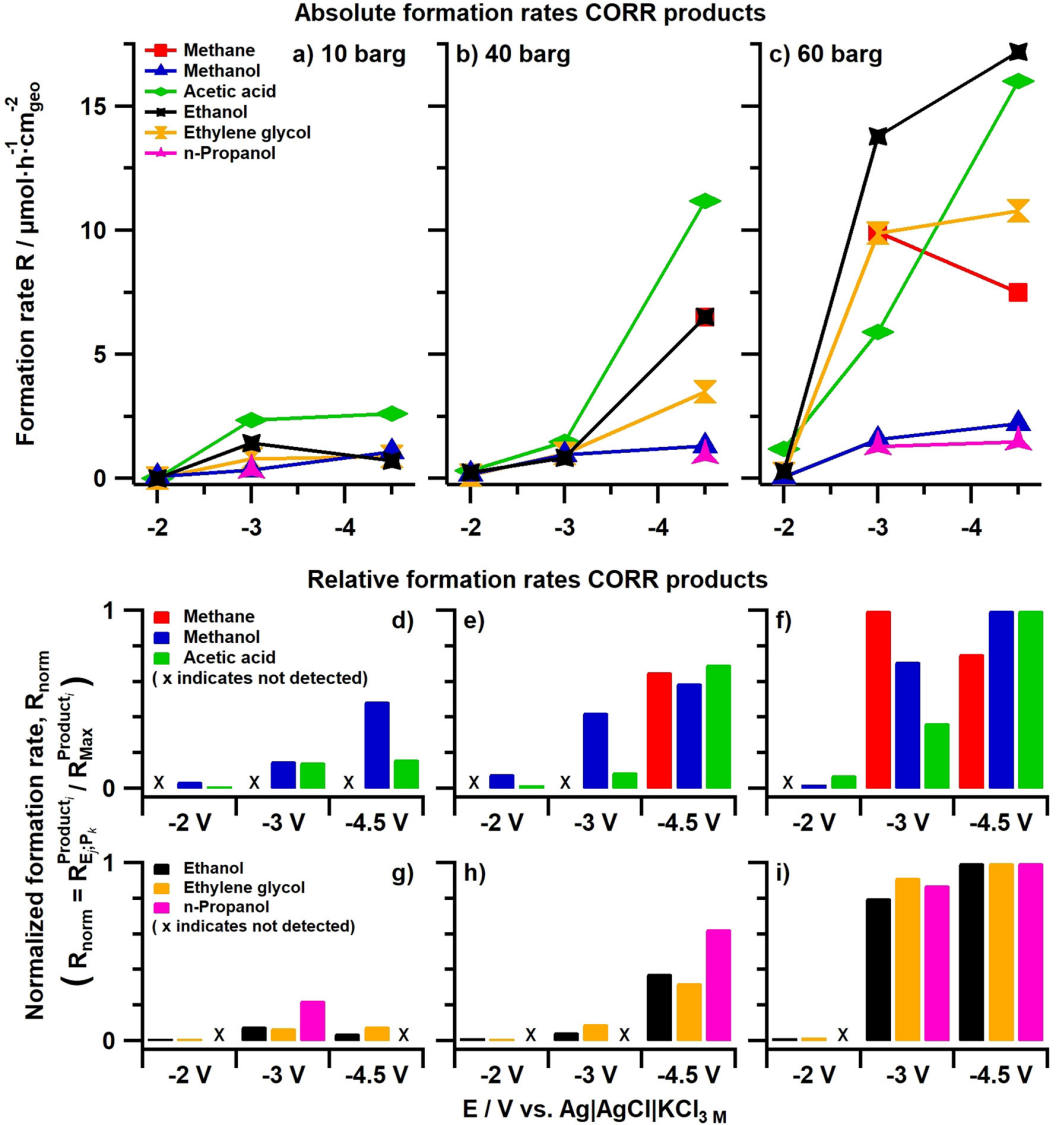
Color‐coded formation rates for CORR products (methane: red, methanol: blue, acetic acid: green, ethanol: black, ethylene glycol: orange, *n*‐propanol: pink) plotted as a function of applied potential (non‐IR corrected) for three different reactant pressures; 10 barg (a, d, and g), 40 barg (b, e, and h) and 60 barg (c, f, and i) expressed in absolute rates (a, b, and c) and relative rates (d, g and e, h and f, i). All axes in a given row are of equal magnitude. Not detected products are marked by an “x” in the subfigures depicting relative rates.

Specifically, the CORR‐related products (Figure 1) comprise a product with carboxylic acid functionality (acetic acid, green), the simplest hydrocarbon (methane, red), and four compounds with alcohol functionality (methanol, ethylene glycol, ethanol, and *n*‐propanol; blue, orange, black, and purple, respectively). Notably, ethylene, which is very commonly observed on copper electrodes,^[1d]^ was not observed. The predominance of oxygenates (excluding methane) agrees with the DFT predictions of Hanselman et al., who computed silver to be a poor catalyst for breaking C−O bonds.^[19]^ Unconventionally, formation rates rather than partial current densities are depicted in Figure 1. This approach allows for directly comparing molar product ratios, which is valuable from a mechanistic point of view considering certain reaction pathways yielding C_2_ species (e.g., Cannizzaro disproportionation^[25]^) would result in equimolar concentrations of particular types of products. Partial current densities are provided in Figures S4 (for CORR products) and S5 (for hydrogen), whilst the overall current response of the system is depicted in Figure S6. Faradaic efficiencies are given in Table S1.

Pressure and potential dependencies for these CORR products can be determined from Figure 1 a–c. Overall, formation rates increase when either the overpotential or CO pressure is increased, although formation rates at 10 barg/−3 V and methane formation at 60 barg/−3 V are exceptions. However, because the products’ formation rates overlap to a considerable degree, these figures can only provide us with general trends. To better distinguish individual trends, each product has been normalized to its highest observed formation rate and is depicted on a per‐pressure basis in Figure 1 d–f (for methane, methanol, and acetic acid) and Figure 1 g–i (for ethanol, ethylene glycol, and *n*‐propanol) for 10, 40, and 60 barg from left to right, respectively. The first group (methane, methanol, and acetic acid) comprises products weakly correlating to pressure, potential, and one another whereas the second group (ethanol, ethylene glycol, and *n*‐propanol) is comprised of products that show fairly straightforward trends that are shared between them.

The behavior of these latter three higher alcohols yields important insights into the C−C formation mechanism since they all exhibit very similar trends: at the lowest applied pressure and potential (10 barg, −2 V) they are just barely detectable. Then, as the potential is decreased (−3 V) their formation rates go through a maximum and subsequently slightly decrease again for higher overpotentials (−4.5 V). Increasing the CO pressure from 10 to 40 barg results in this maximum disappearing, with observed relative formation rates increasing rapidly as higher overpotentials are applied. However, this potential dependency becomes weaker as the pressure is increased further, with more moderate increases of ca. 5–25 % observed between successively more negative potentials at CO pressures of 60 barg.

Exhibiting such strong similarities in their potential and pressure dependency indicates commonalities in their formation mechanism, separate from the pathway via which methanol and acetic acid form (to be discussed later). The absence of ethylene (which cannot be explained by insufficient hydrogen coverage, considering the still high rate of H_2_ formation) in concert with the comparable behavior of ethanol and *n*‐propanol is especially interesting. Namely, this observation makes it unlikely that the coupling of CO and ethylene (“hydroformylation”) is responsible for the formation of C_3_ products on silver, as hypothesized to occur on copper by Ren et al.^[26]^ Instead, acetaldehyde, being both reactive and difficult to detect via standard NMR techniques (especially in alkaline media),^[27]^ is known to only reduce to ethanol and not ethylene (on copper).^[28]^ Its high reactivity would facilitate further reduction rather than desorption. This possibility would agree with recent work by Xu et al. who showed that propanol is formed on copper via the coupling between CO and a surface‐bound methylcarbonyl, an intermediate which is one hydrogen short of acetaldehyde.^[28b]^ This latter observation agrees well with DFT calculations conducted by Hanselman et al., who propose ethanol formation takes place via a surface‐bound acetaldehyde species.^[19]^

The fact that both ethylene glycol and ethanol are observed and exhibit similar behavior proves that silver is capable of breaking one of the C−O bonds in a molecule comprised of two carbon atoms containing two C−O bonds. However, the absence of ethylene shows that silver is indeed a poor catalyst for breaking the final C−O bond, as predicted by DFT calculations. From these observations, our results suggest that an oxygenated intermediate, probably surface‐bound methylcarbonyl (as proposed by Hanselman et al. and Xu et al.),[^19^, ^28b^] is involved in the formation of ethanol, as well as in the coupling with adsorbed CO to lead to the formation of *n*‐propanol (through propanal).

Additional insights regarding C−C coupling on silver can be derived from the behavior of the other “group” of products (methane, methanol, and acetic acid) whose trends with regards to potential, pressure, and one another are more inconsistent. Of these, the methane “trends” disagree with all other observed CORR products. The most notable observation that can reasonably be made is that it is more prevalent at increased CO pressures and more cathodic potentials. More important are methanol and acetic acid, as they exhibit some similarities although their correlation is much weaker than the previously discussed alcohols. Comparing these products, we find that methanol generally exhibits higher relative formation rates than acetic acid at lower overpotentials, and for all investigated potentials in the case of 10 barg of CO pressure. However, when the pressure is increased (from 10 to 40 or 60 barg), relative acetic acid formation rates start to become very similar to those of methanol formation for the most cathodic potentials investigated (−4.5 V). This results from the fact that methanol formation rates are relatively invariant with potential and pressure, whereas acetic acid is strongly influenced by both of these parameters. (This observation that acetic acid formation remains strongly potential dependent also at increased pressures is what makes its behavior different from the previously discussed “alcohol group” as they exhibit much weaker relative increases in formation rate with potential at 60 barg of CO.)

The strong pressure dependency of acetic acid suggests that CO is involved in its formation. Furthermore, the fact that this dependency persists even at elevated reactant pressures signifies that the C−C coupling step for its formation has a significant barrier. Additionally, the (weak) correlation observed between methanol and acetic acid can be interpreted as them sharing a common intermediate. Hence we speculate there may exist a pathway where CO couples with a methanol‐like moiety to form acetic acid. Some plausibility for this hypothesis can be derived from the existence of a rhodium‐catalyzed industrial process for acetic acid synthesis involving the carbonylation of methanol called the Monsanto process.^[29]^ However, we emphasize that the most important observation from Figure 1 is that the pathway for the formation of acetic acid differs from the pathway via which ethanol, ethylene glycol, and *n*‐propanol are formed.

In summary, high‐pressure CO electroreduction experiments reveal that silver is capable of further reducing carbon monoxide if the CO surface coverage is sufficiently high, with the total production rates of C_2+_ CORR products (ethanol, ethylene glycol, and propanol) increasing as the pressure is increased. Contrary to one literature report,^[15b]^ ethylene formation was not observed in this work. The fact that silver is capable of reducing CO to ethanol but not to ethylene is in agreement with DFT calculations.^[19]^

The comparable potential and pressure dependence of the formation of ethanol, *n*‐propanol, and ethylene glycol indicates a commonality in their formation pathways. An oxygenated surface species is likely to be the shared intermediate between ethanol and *n*‐propanol, and this species is likely to be one hydrogen short of acetaldehyde, as suggested by Hanselman et al. and Xu et al.[^19^, ^28b^] We propose it is the coupling of this species with adsorbed CO that is responsible for the formation of propanal, which is then further reduced to *n*‐propanol, as opposed to a reaction between a surface‐bound ethylene molecule and carbon monoxide (Figure 2).

**Figure 2:**
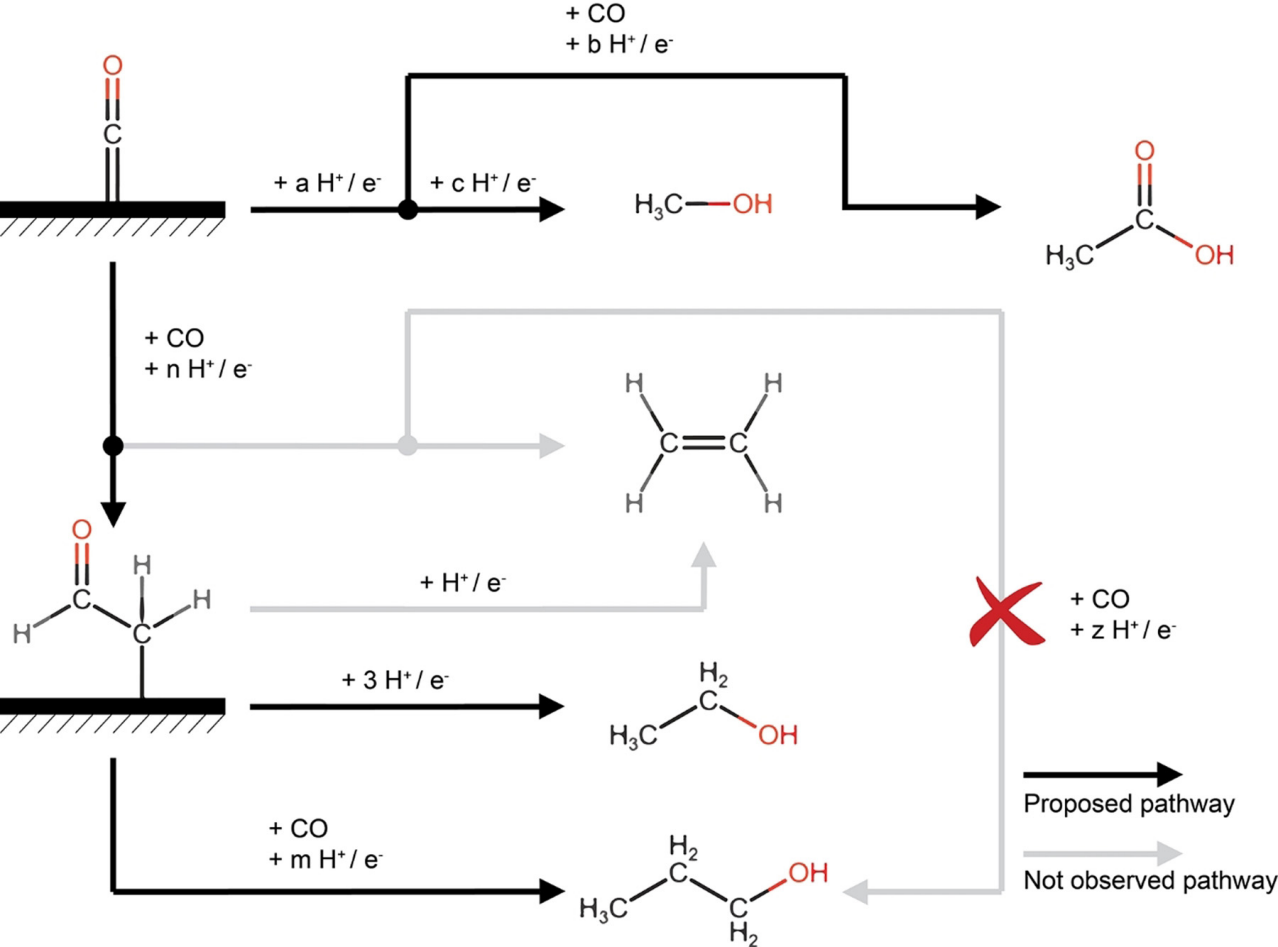
Proposed mechanistic pathway based on literature and the products (and their trend similarities) observed in this study.

If the CO coverage is sufficiently high, as can be achieved by increasing CO pressure, the product spectrum of silver starts to resemble that of copper under CO2RR conditions.^[5]^ However, the formation rates for CORR products on silver are orders of magnitude lower than what is observed on copper, making detecting minority products beyond the scope of this work. The main difference between the two systems seems twofold. Firstly, due to the rather unfavorable adsorption energy of CO, silver has the propensity for desorbing CO rather than reducing it further, even though thermodynamically speaking it is capable of doing so. Secondly, due to silver being a poor catalyst for breaking C−O bonds,^[19]^ no ethylene (nor ethane) formation is observed although the rest of the products observed compare favorably with copper‐catalyzed CO_(2)_ reduction.

## Conflict of interest

The authors declare no conflict of interest.

## Supporting information

As a service to our authors and readers, this journal provides supporting information supplied by the authors. Such materials are peer reviewed and may be re‐organized for online delivery, but are not copy‐edited or typeset. Technical support issues arising from supporting information (other than missing files) should be addressed to the authors.

Supporting Information
