# Supplementary material for: High‐Pressure CO Electroreduction at Silver Produces Ethanol and Propanol
Source: Angew Chem Int Ed Engl. 2021 Aug 25;60(40):21732–6. doi: 10.1002/anie.202108902 (PMC8518692; doi:10.1002/anie.202108902)

Angewandte Chemie
Eine Zeitschrift der Gesellschaft Deutscher Chemiker

Supporting Information

High-Pressure CO Electroreduction at Silver Produces Ethanol and Propanol

*Stefan J. Raaijman, Maarten P. Schellekens, Paul J. Corbett, and Marc T. M. Koper**

anie_202108902_sm_miscellaneous_information.pdf

Supporting experimental

Chemicals. All solutions were prepared by dissolving appropriate amounts of chemicals, used as received unless otherwise indicated, in high purity Milli-Q water (Millipore, 18.2 MΩ·cm). All experiments were conducted in 300 mL 0.5 M KOH (99.95%, Alfa Aesar) electrolyte purged and pressurized with carbon monoxide (Linde, 4.7), whilst the counter electrode compartment was purged with nitrogen (Linde, 2.0).

Electrochemistry. Potentials were controlled via either a Solartron (Energylab XM with 2A booster) or Autolab (PGSTAT302N) potentiostat in a three-electrode configuration. Solution resistances were determined via impedance before the start of the experiment and at regular intervals during the measurement, but reported potential values are not corrected for ohmic drop.

High-pressure electrochemical cell and product detection. A home-made three-compartment electrochemical cell designed to fit inside an autoclave (Premex, Hastelloy C22, 600ml) was used for all experiments reported in this work (Figure S1). The autoclave had a total of three gas inlets (CE compartment, WE compartment topside (not used) and WE backside) and two gas outlets (CE and WE compartment outlets). The inlets of the autoclave were attached to calibrated mass flow controllers (Brooks, Delta Smart II) connected to their respective gas bottles, whilst the outlet was attached to a back-pressure regulator (Equilibar) to regulate pressure inside the reaction chamber and allow for constant flow. Outflows of excess gases for counter and working compartments were united downstream, but the existence of a constant net positive flux for both compartments prevented mixing at points located upstream. After, exhaust gas was led through a dehumidifier (Perma Pure, MD-050-24S-2) prior to being introduced into a gas chromatograph (G.A.S, CompactGC 4.0).

The electrochemical cell was comprised of two sections: a bottom and a top half. The lower half contained the electrochemical cell and consisted of a cylindrical PTFE beaker that was machined to precisely fit the bottom of the autoclave. The top half of the cell was affixed to the top of the autoclave, and housed a series of machined plastic pieces that extended down into the PTFE beaker upon assembly. Specifically, the top contained a total of five elements: separate housings for both the counter (Figure S1, A) and reference electrodes (Figure S1, C), a machined component capable of flowing gases along the back of the working electrode into the reaction vessel (Figure S1, D), a vertical stirring rod (Figure S1, B), and a final piece of 3D printed solid plastic (3D printed with Form labs 2 SLA 3D printer using FLGPC02 resin) that was shaped so as to fill the empty spaces in-between the other components above the electrolyte level (Figure S1, yellow sections). Electrical contact with the outside was established via pass-through Swagelok fittings located in the top of the autoclave.

The counter electrode (platinum mesh, 99.9%, Alfa Aesar) was housed in a rectangular enclosure made out of PEEK with an internal volume of ca. 20 mL. The anolyte was isolated from the rest of the cell via an anion exchange membrane (AEM, Fumasen, FAB-PK-130) of ca. 7 cm^2. The anolyte was constantly purged with nitrogen to facilitate mixing and the removal of bubbles, whilst also serving as an internal standard for gas quantification. The membrane separating the two compartments was replaced daily, or alternatively after each experiment (for experiments >12 h).

A home-made Ag/AgCl wire in contact with KCl solution made up the reference electrode. Prior to each experiment the AgCl layer was renewed by submerging the silver wire (99.9%, Alfa Aesar) in a 0.1 M HCl solution (Ph. Eur., Merck) and applying a current of +20 mA for 20 seconds vs. a Pt wire to form a silver chloride overlayer, which is the electrochemical equivalent of some commonly employed[1] chemical treatments to generate a AgCl layer. Then, the wire was thoroughly rinsed with Milli-Q water, after which it was submerged in a machined Luggin capillary made from Kel-F containing ca. 10 mL AgCl saturated (0.16 M) 3 M KCl solution (Merck). The tip of the Luggin capillary was located in close proximity to the bottom-half of the working electrode surface (ca. 1 mm away) and terminated with a PEEK frit with 2 µm pores (Idex, A-710) to separate the working electrolyte from the reference solution.

The working electrode assembly was comprised of an open-ended hollow rod that allowed for gases to be introduced to the reaction vessel. Electrical contact with the working electrode was established through the backside of the WE via a circular titanium plate (connected to a lead) that had flow channels machined into it to allow gases to reach the backside of the electrode. However, no means of closing the lower end of the tube were present. Thus, gases could also flow past the backside of the electrode and out of the bottom tube into the electrolyte. A silver GDE (Dioxide Materials, Ag/ionomer coating on carbon paper) was cut to size and used as received, with a total geometric area of 1 cm^2 exposed to the electrolyte. The working electrode was replaced for each individual measurement.

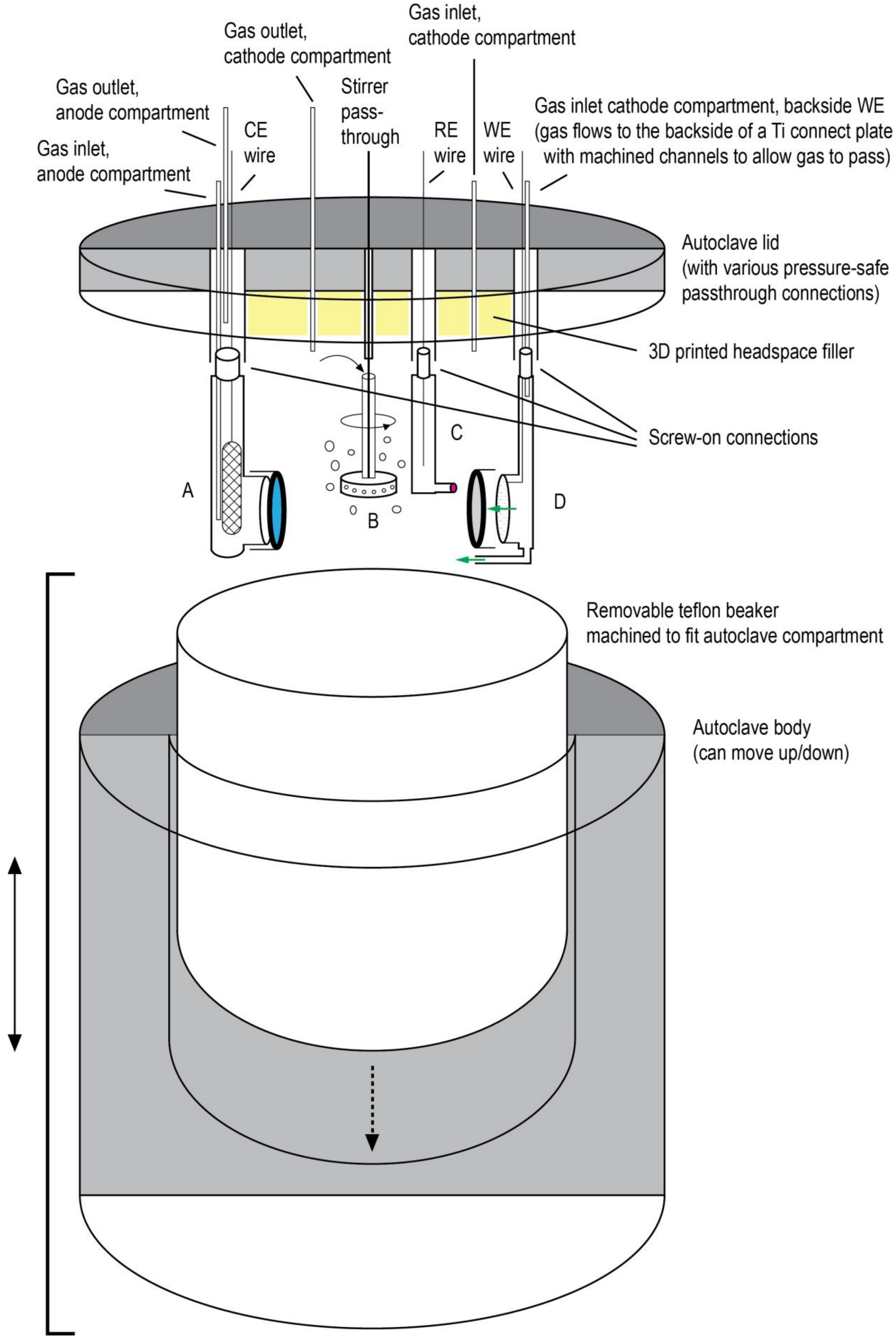

Figure S1. Schematic of the high-pressure setup.

IR drop determination. Solution resistances were determined at the start of each experiment via impedance spectroscopy near the open circuit potential and assumed to be equal to the measured real impedance at 10 kHz[2], which varied between 4.8 and 5.5 Ω for all experiments reported in this work. Potentials as we report them (V vs. Ag|AgCl|KCl (3 M)) can be converted to the reversible hydrogen electrode (RHE) scale via eq. 1, and the ohmic resistance compensated to obtain the 'real' interfacial potential via eq. 2 where conventional signage for cathodic (negative) and anodic (positive) current applies. Using a standard potential of $E_{Ag|AgCl\ (3\ M)}$ = +0.21 V vs. SHE, a pH of 13.7 for our 0.5 M KOH electrolyte, an R value of 5 Ω, and -0.5 A of current at the most negatively applied potential of -4.5 V (see Figure S6, considering 1 cm^2 of area), we obtain a 'real' potential of ca. -1 V vs. RHE.

$$V\ vs.RHE = V\ vs.Ag|AgCl|KCl_{3\ M} + E^{3\ M\ Cl^-}_{Ag|AgCl}(V) + pH * 59.1 * 10^{-3}(V) \quad (1)$$

$$V_{real} = V_{applied} - i\ (A) * R_u(\Omega) \quad (2)$$

Cleaning. Organic and metallic contaminants were removed from the cell body by submerging in acidified (0.5 M H_2SO_4, Ph. Eur, Merck) permanganate solution (1 g/L $KMnO_4$, Baker Chemicals) for a minimum of 12 h, which was rinsed with dilute piranha solution followed by thorough rinsing with Milli-Q water prior to use. The upper compartment housing the electrode compartments were cleaned by storing in Milli-Q water and rinsing them prior to use. All parts of the cell in direct contact with electrolyte were made of various chemically inert polymers (PTFE, PEEK or Kel-F), to prevent metal ion leaching by the highly alkaline electrolyte.

Product analysis. The gaseous cell exhaust was sampled at regular intervals (1-6 times/hr) via gas chromatography, with the sampling frequency depending on reactant gas inflow (15-150 mL_n/min) with lower flows (and less frequent sampling intervals) being used for experiments that necessitated longer reaction times (i.e. lower currents). The addition of a known flow of inert gas (nitrogen) was used as an internal standard to calculate the flows of the individual components. After each experiment, a liquid sample was taken from the catholyte and analyzed via NMR with DMSO as an internal standard similar to the procedure as described by Kuhl *et al*.[2]

Control experiments to verify product origin

Two measurements were performed so as to verify that the origin of products is electrochemical in nature, with the electrolyte composition investigated via NMR for products (Figure S2). Firstly, the reactor was set up identically as discussed previously and pressurized to 40 barg of CO, including continuous gas flow. However, no potential was applied to the WE. This condition was kept for >60 hours, and the 'electrolyte' was analyzed for products (Figure S2a). In this instance, two liquid (and no gaseous) products discussed in the main text were observed: formic acid and acetic acid. Formic acid formation from CO + OH^- has been addressed in the main text, but the fact that we see acetic acid is peculiar. Although its formation rate is much below what we observe in the presence of an applied potential (it is about 35% of the lowest formation rate detected in this work i.e., for 10 barg of CO at -2 V), one would not expect its formation at all in absence of an applied potential. This trace of acetic acid may originate from a reaction between CH_4 and CO_2.[3] Carbon dioxide will be present in minor quantities due to the reverse water-gas shift reaction (CO + H_2O <=> CO_2 + H_2), whilst methane is present as an impurity in the CO gas (and we also observe in the GC). Although this reaction is unfavorable, and the quantities of required reactants are low, it may serve as an explanation for the trace of acetic acid we observe, even in the absence of an applied potential.

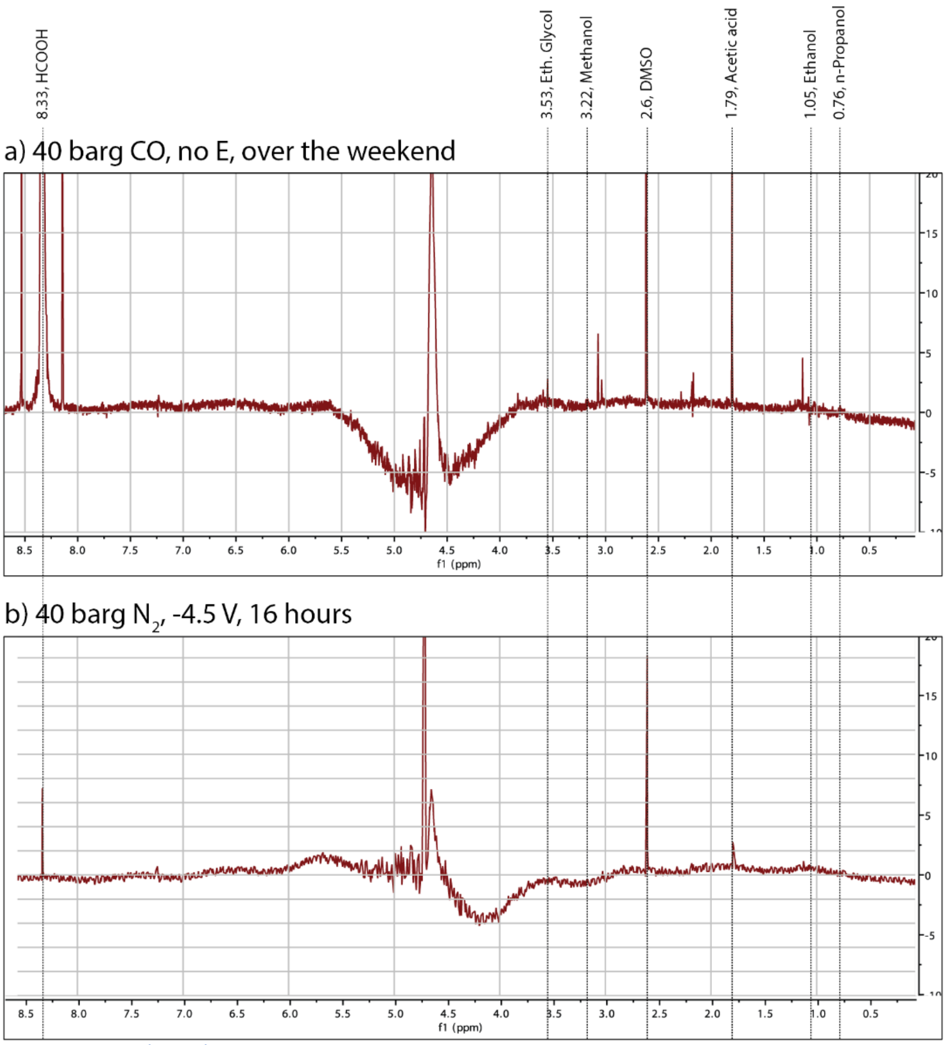

Figure S2. NMR spectra control experiments

As for the second control experiment, we applied -4.5 V under 40 barg of N_2. In this instance, no appreciable quantities of any kind of product other than hydrogen were observed (although a small spike representing formic acid can be seen, likely originating from traces of CO left in the dead spots of the system as we also found from GC, but its concentration is insignificant).

Product formation rates for non-CORR-related (majority) products

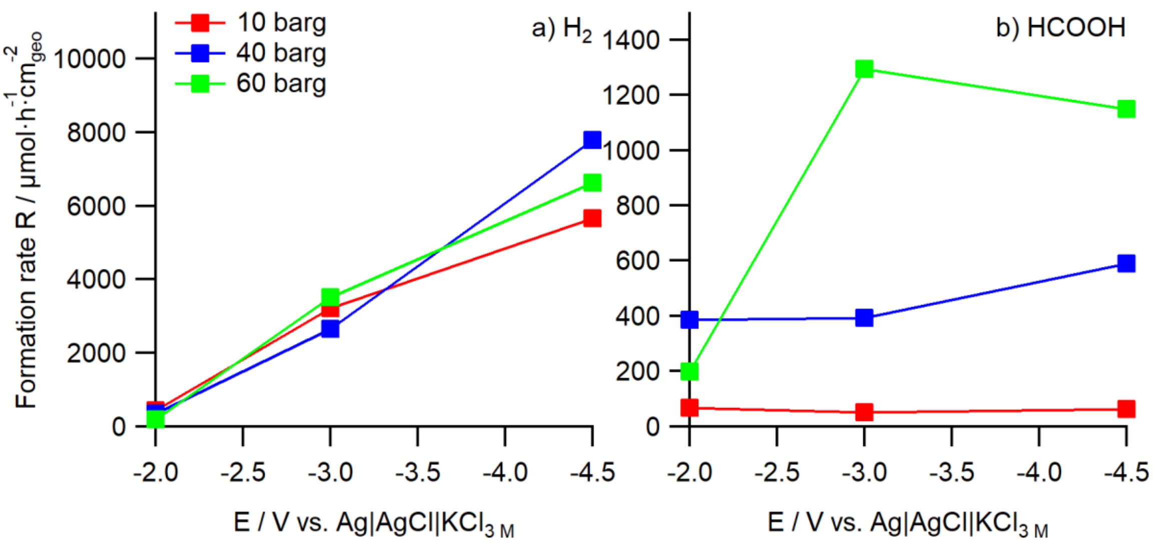

Figure S3. Area-normalized formation rates for non-CORR products.

Electrochemical response of the system

Partial current densities for the CORR-related products discussed in the main text are depicted in Figure S4, and the partial current density for hydrogen is shown in Figure S5. Beware of the difference in scale between the two figures (µA vs mA for Figures S4 and S5, respectively). Total system current densities for the various investigated conditions are depicted in Figure S6.

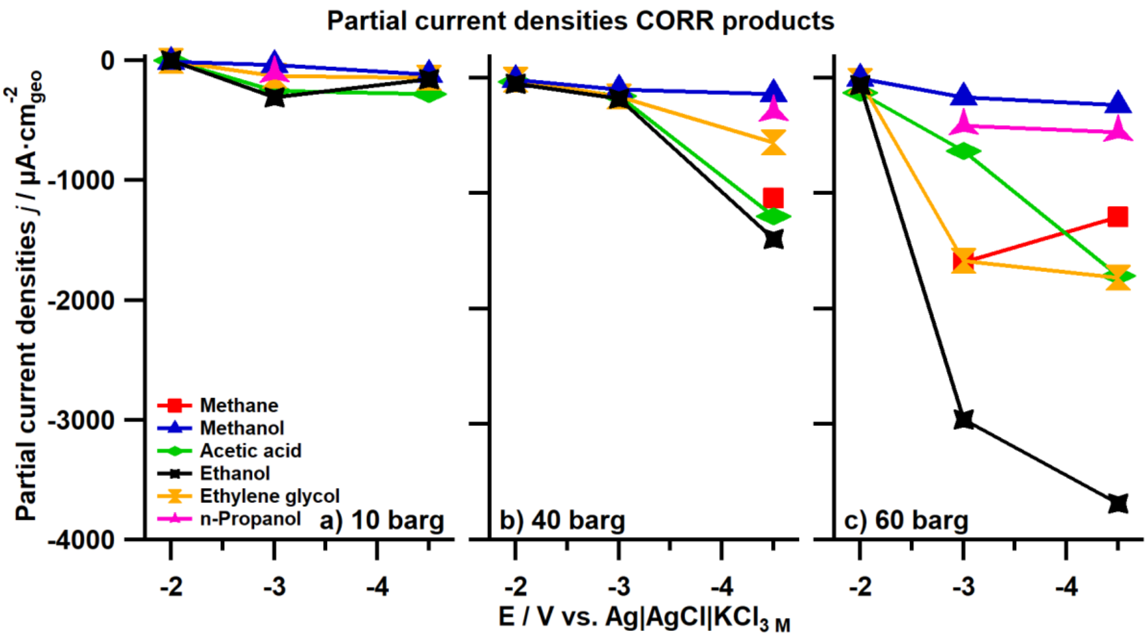

Figure S4. Partial-current density equivalent plot of Figure 1 in the main text.

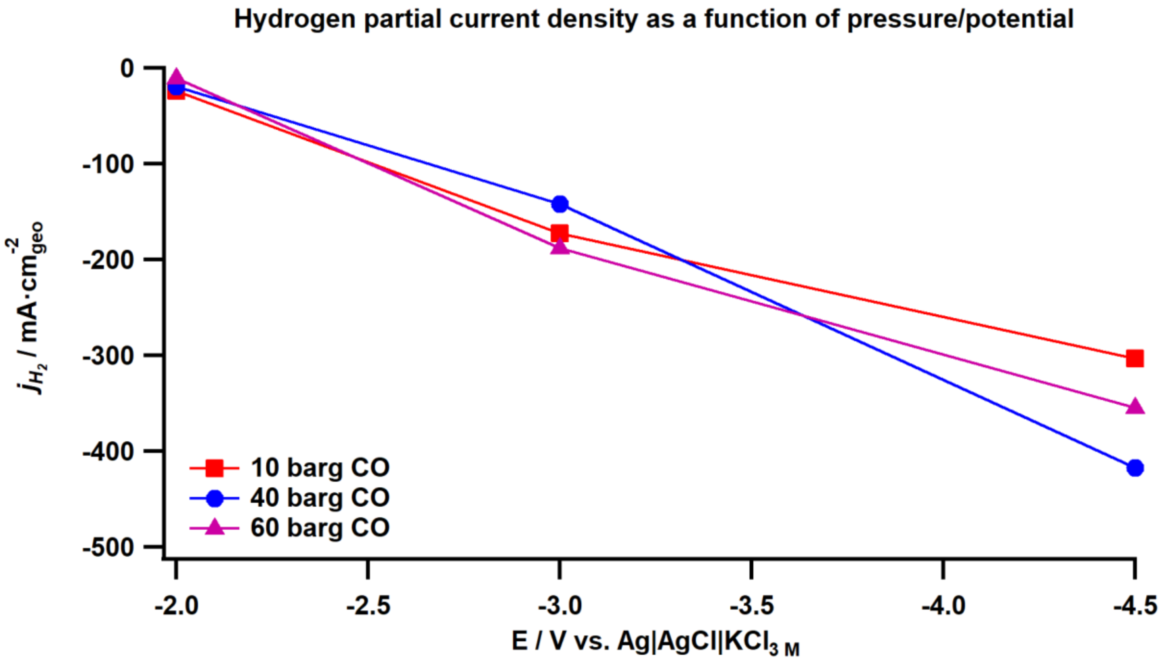

Figure S5. Pressure-dependent partial current density of hydrogen.

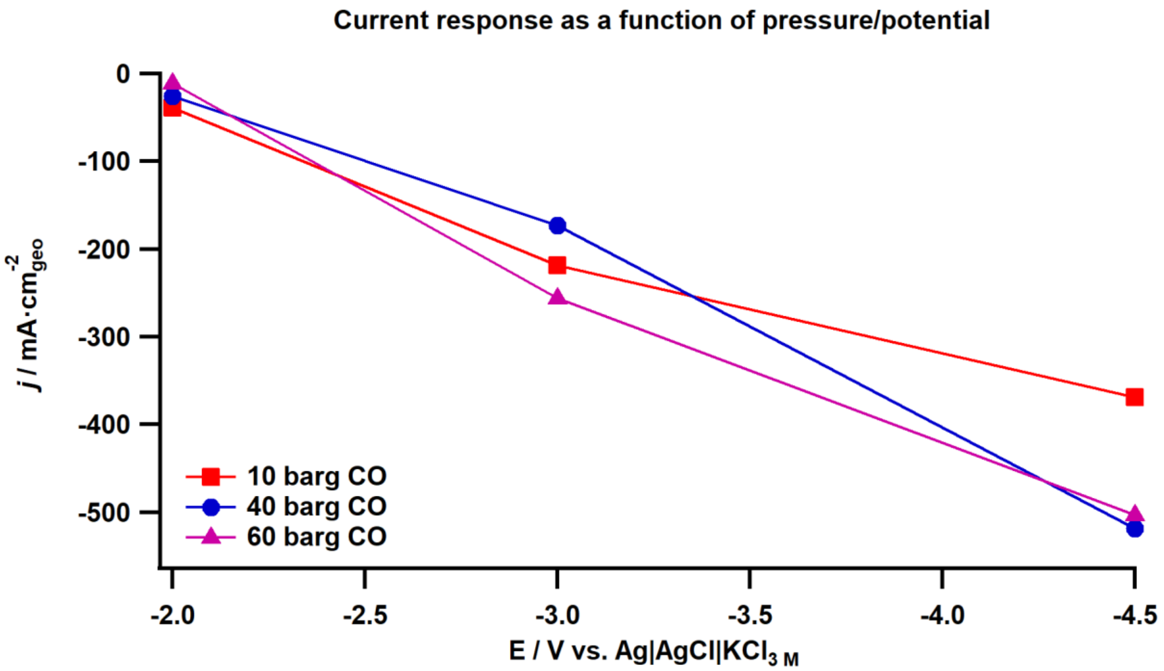

Figure S6. Total current densities for the various investigated conditions.

Faradaic efficiencies for the measurements discussed in the main text are provided in Table S1. Two main points stand out. Firstly, CORR products are the minority species, which is reflected in low faradaic efficiency values for these products. Secondly, not all current in the system is accounted for. The latter we explain by the fact that our reactor at the time of measuring did not have a perfect seal (an issue that has since been addressed), allowing part of the hydrogen to escape the system. Considering that hydrogen accounts for the vast majority of the current, a loss of hydrogen (as it is very fugacious) through to a small leak in the system has large repercussions for the total faradaic efficiency. However, this issue is expected to have little to no influence on the identities and quantities of solution-phase products, as they are not meaningfully present in the gas phase due to their lower vapor pressure, and therefore should not significantly influence our discussion. Regarding methane, which would be affected, it is only sporadically observed (to rise above the always present baseline due to trace impurities in the CO bottle) and due to this does not match any trend, even if there is a larger error bar for those instances where it is observed.

Table S1. Faradaic efficiencies of observed reaction products during CORR on Ag.

		Faradaic efficiencies (%):							Sum (%):
		Ethylene glycol	Methanol	Acetic acid	Ethanol	n-Propanol	H2	CH4	
10 barg	-2 V	0.003	0.023	0.001	0.002	0.000	60.960	0.000	60.990
	-3 V	0.058	0.017	0.116	0.139	0.049	79.078	0.000	79.457
	-4.5 V	0.039	0.032	0.076	0.042	0.000	82.287	0.000	82.476
40 barg	-2 V	0.077	0.074	0.135	0.203	0.000	73.742	0.000	74.231
	-3 V	0.094	0.059	0.092	0.104	0.000	82.092	0.000	82.442
	-4.5 V	0.108	0.027	0.231	0.270	0.057	80.544	0.201	81.439
60 barg	-2 V	0.283	0.051	1.175	0.584	0.000	96.618	0.000	98.710
	-3 V	0.620	0.066	0.247	1.154	0.161	73.374	0.623	76.246
	-4.5 V	0.345	0.047	0.341	0.732	0.094	70.505	0.239	72.303

Summaries of literature-reported C-C bond formation mechanisms

Reaction schemes summarizing the various proposed formation pathways for C2 and C3 products (in A3 format) are provided at the very end of the supporting information. Subsets of mechanisms proposed by the same groups have been combined into single schemes using color coding to distinguish separate publications. Different reaction mechanisms are indicated by different letters (going from A to J), combined with either -C2 or -C3 to denote if a proposed pathway leads to C2 or C3 products. Mechanisms with the same lettering are proposed by the same group(s) of people (e.g., A-C2 and A-C3 would represent the mechanism by which C2 and C3 products are formed, respectively, as proposed by the same group). References to the various publications are provided in the individual schemes, together with the color coding used for each scheme (i.e., which color belongs to which reference). Proposed reaction schemes that are similar in nature (e.g., having similar types of intermediates, overlapping steps etc.) have been grouped together as follows for C2 formation schemes: A-B, C-E, F-H, and I. The proposed mechanism J-C3 does not have a corresponding C2 mechanism, though they refer to the work represented by the mechanisms A-C2 and A-C3.

References

[1] a) G. A. East, M. A. del Valle, *J Chem Educ* **2000**, *77*, 97; b) R. Barlag, F. Nyasulu, R. Starr, J. Silverman, P. Arthasery, L. McMills, *J Chem Educ* **2014**, *91*, 766-768.

[2] K. P. Kuhl, E. R. Cave, D. N. Abram, T. F. Jaramillo, *Energy Environ Sci* **2012**, *5*, 7050-7059.

[3] E. M. Wilcox, G. W. Roberts, J. J. Spivey, *Catal Today* **2003**, *88*, 83-90.

A-C_2

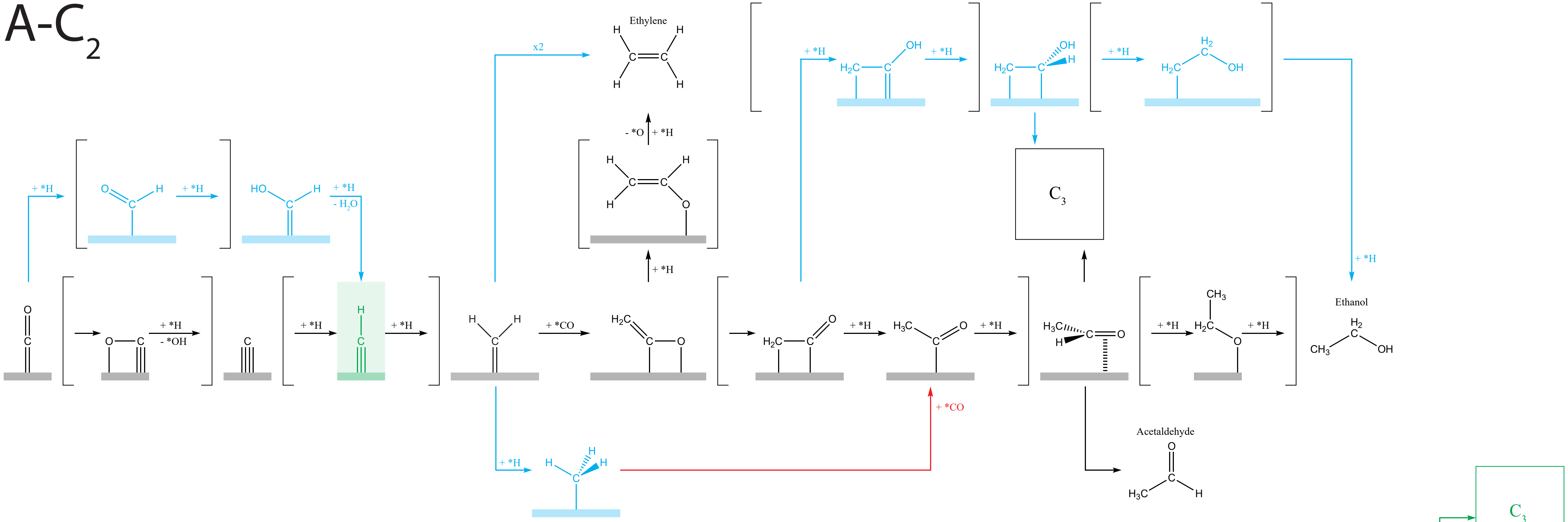

Blue Cu(poly): Hori, Y. (Takahashi, R.), Formation of hydrocarbons in the electrochemical reduction of carbon dioxide at a copper electrode in aqueous solution. J Chem Soc, Faraday Trans 1 1989, 85 (8), 2309-2326.
Red Cu(100) terraces with (110) steps: Wang, X. (Strasser, P), Morphology and mechanism of highly selective Cu(II) oxide nanosheet catalysts for carbon dioxide electroreduction. Nat Commun 2021, 12 (1), 794.
Green Cu(poly): Ikeda, S. (Noda, H.), Electrochemical Mass Reduction of Carbon Dioxide using Cu-Loaded Gas Diffusion Electrodes II. Proposal of Reaction Mechanism. Denki Kagaku oyobi Kogyo Butsuri Kagaku 1996, 64 (1), 69-75.
Black Cu(poly): Hori, Y. (Murata, A.), Electrochemical Reduction of CO at a Copper Electrode. J Phys Chem B 1997, 101 (36), 7075-7081.

B-C_2

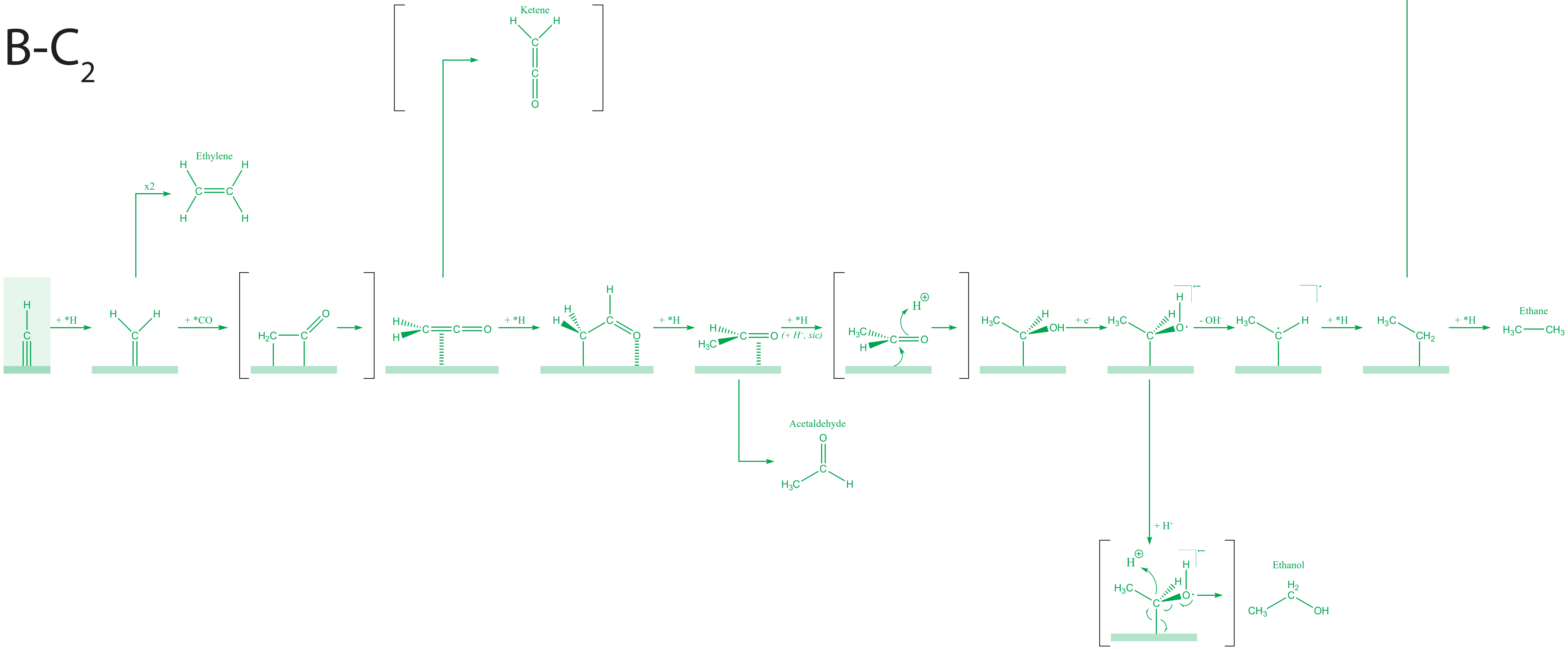

C-C$_2$

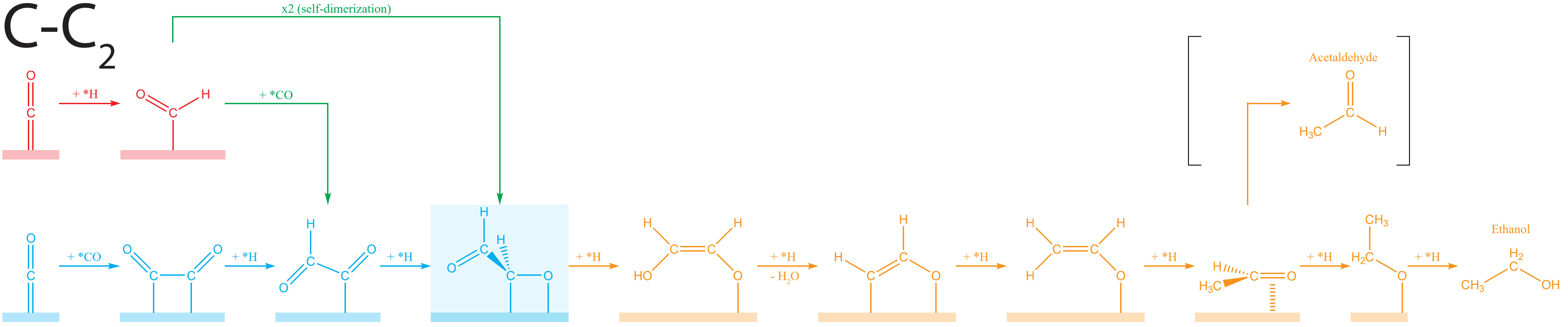

Orange Cu(211): Bertheussen, E. (Chorkendorff, I), Acetaldehyde as an Intermediate in the Electroreduction of Carbon Monoxide to Ethanol on Oxide-Derived Copper. Angew Chem Int Ed 2016, 55 (4), 1450-1454.
Red Cu(211): Peterson, A. A. (Nørskov, J. K.), How copper catalyzes the electroreduction of carbon dioxide into hydrocarbon fuels. Energy Environ Sci 2010, 3 (9), 1311-1315.
Green Cu(211): Montoya, J. H. (Nørskov, J. K.), Insights into C-C Coupling in CO2 Electroreduction on Copper Electrodes. ChemCatChem 2013, 5 (3), 737-742.
Blue Cu(100) & Cu(111): Montoya, J. H. (Nørskov, J. K.), Theoretical Insights into a CO Dimerization Mechanism in CO2 Electroreduction. J Phys Chem Lett 2015, 6 (11), 2032-2037.
Black Cu(poly): Kuhl, K. P. (Jaramillo, T. F.), New insights into the electrochemical reduction of carbon dioxide on metallic copper surfaces. Energy Environ Sci 2012, 5 (5), 7050-7059.

D-C$_2$

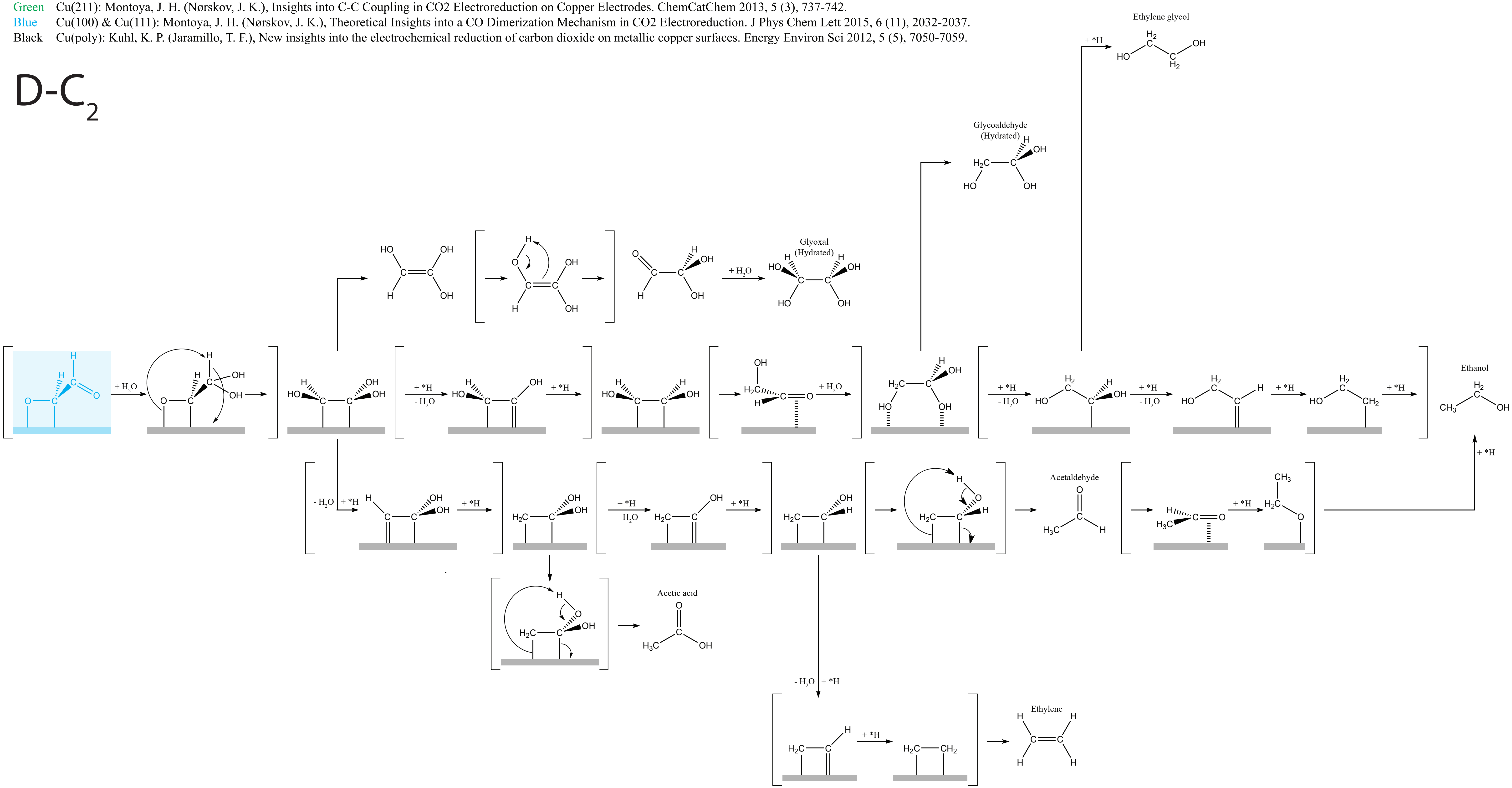

E-C_2

Blue Cu(poly): Kuhl, K. P. (Jaramillo, T. F.), New insights into the electrochemical reduction of carbon dioxide on metallic copper surfaces. Energy Environ Sci 2012, 5 (5), 7050-7059.

Black Cu(poly): Shah, A. H. (He, T.), Revisiting Electrochemical Reduction of CO2 on Cu Electrode: Where Do We Stand about the Intermediates? J Phys Chem C 2018, 122 (32), 18528-18536.

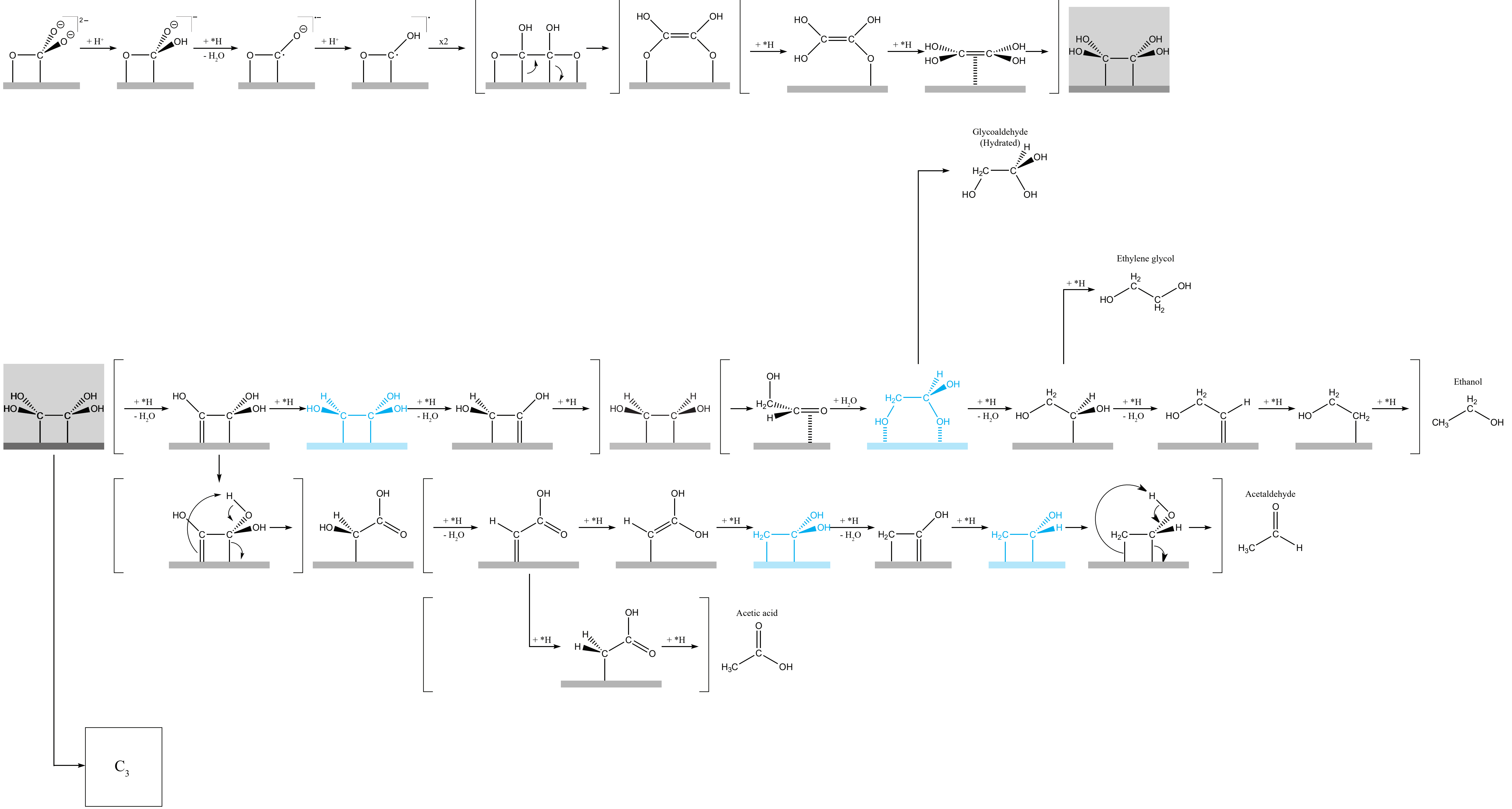

F-C_2

Green Cu(100): Calle-Vallejo, F. (Koper, M. T. M.), Theoretical Considerations on the Electroreduction of CO to C2 Species on Cu(100) Electrodes. Angew Chem Int Ed 2013, 52 (28), 7282-7285.
Red Ag, Cu & Au(100): Hanselman, S. (Calle-Vallejo, F.), Computational Comparison of Late Transition Metal (100) Surfaces for the Electrocatalytic Reduction of CO to C2 Species. ACS Energy Letters 2018, 3 (5), 1062-1067.
Blue Cu(211): Liu, X. (Chan, K), pH effects on the electrochemical reduction of CO(2) towards C2 products on stepped copper. Nat Commun 2019, 10 (1), 32.

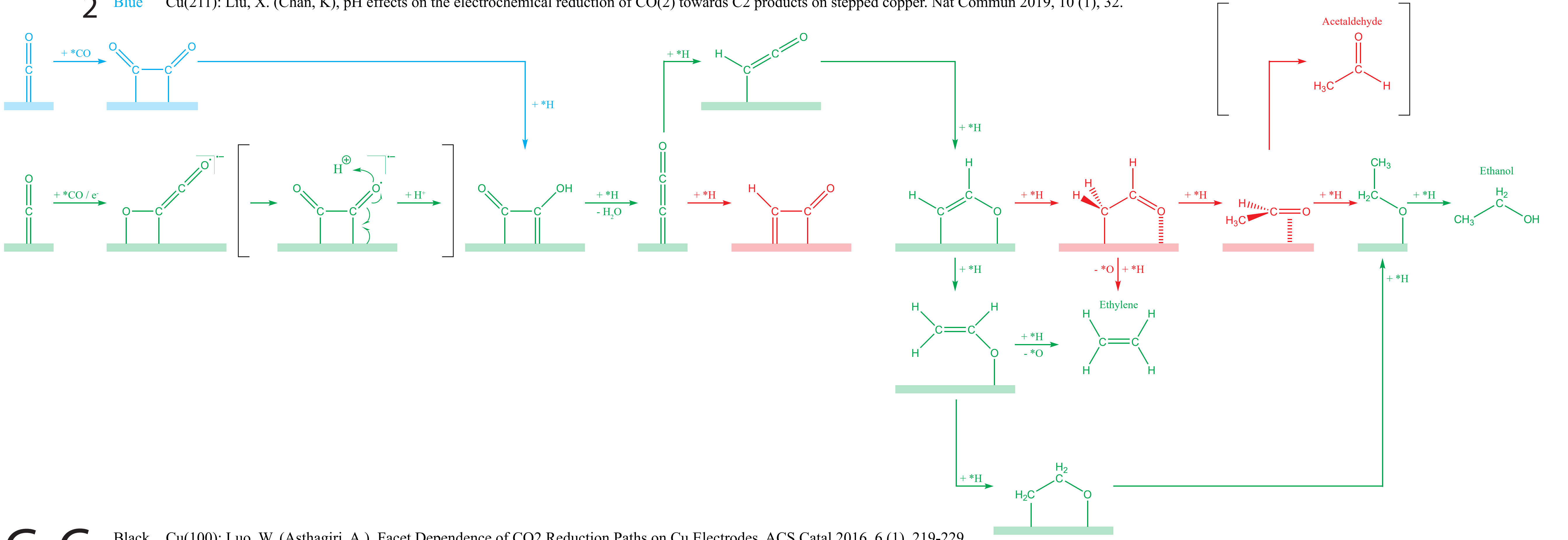

G-C_2

Black Cu(100): Luo, W. (Asthagiri, A.), Facet Dependence of CO2 Reduction Paths on Cu Electrodes. ACS Catal 2016, 6 (1), 219-229.
Orange Cu(111): Nie, X. (Asthagiri, A.), Reaction mechanisms of CO2 electrochemical reduction on Cu(1 1 1) determined with density functional theory. J Catal 2014, 312, 108-122.

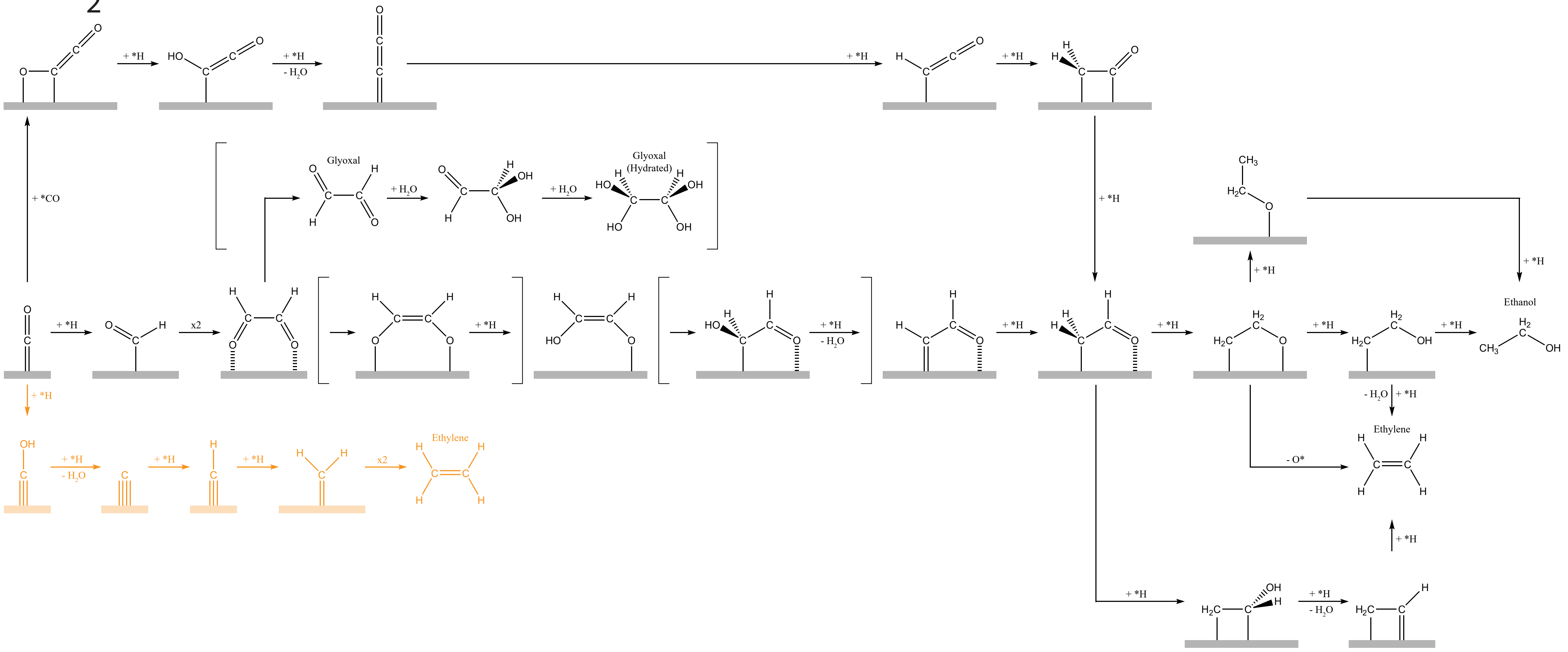

H-C2

Black Cu(100): Garza, A. J.; Bell, A. T.; Head-Gordon, M., Mechanism of CO2 Reduction at Copper Surfaces: Pathways to C2 Products. ACS Catal 2018, 8 (2), 1490-1499.
Red Only Cu(100) and only at low overpotential

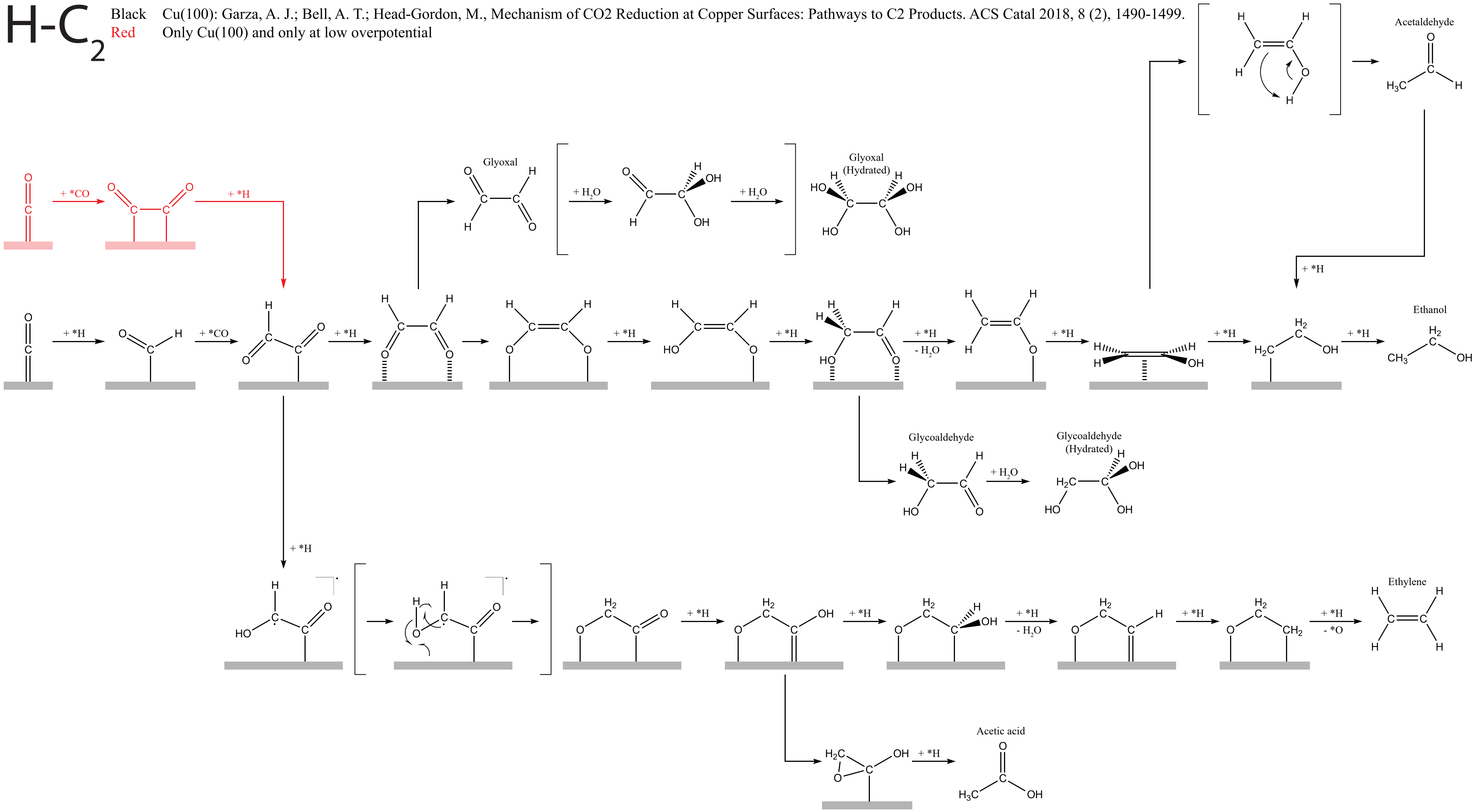

i-C_2

Red Cu(100): Cheng, T. (Goddard, W. A.), Full atomistic reaction mechanism with kinetics for CO reduction on Cu(100) from ab initio molecular dynamics free-energy calculations at 298 K. PNAS 2017, 114 (8), 1795-1800.
Blue Cu(100): Lum, Y. (Ager, J. W.), Electrochemical CO Reduction Builds Solvent Water into Oxygenate Products. J Am Chem Soc 2018, 140 (30), 9337-9340.
Black Cu(100): Xiao, H. (Goddard, W. A.), Atomistic Mechanisms Underlying Selectivities in C1 and C2 Products from Electrochemical Reduction of CO on Cu(111). J Am Chem Soc 2017, 139 (1), 130-136.

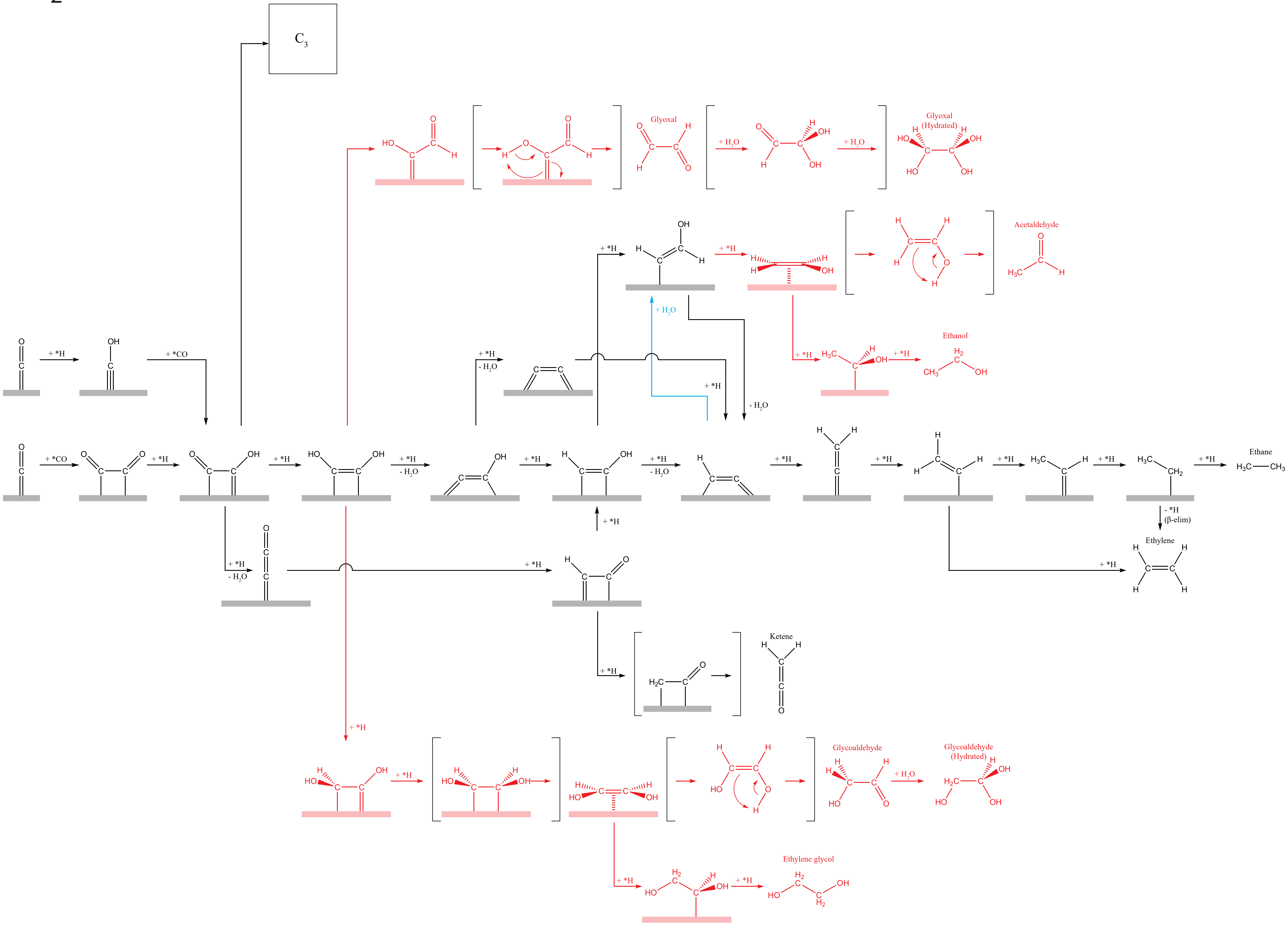

Overview of proposed C-C coupling steps leading to C_3

A-C_3

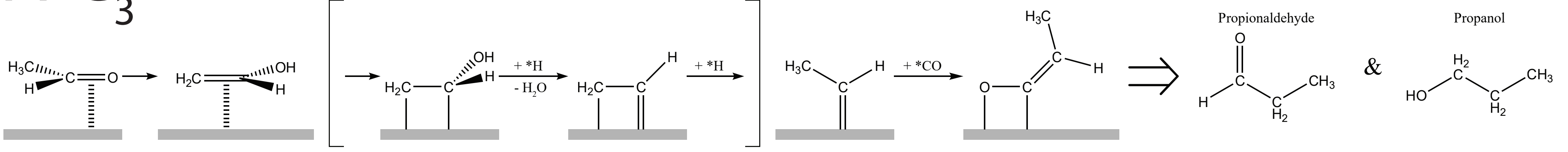

B-C_3

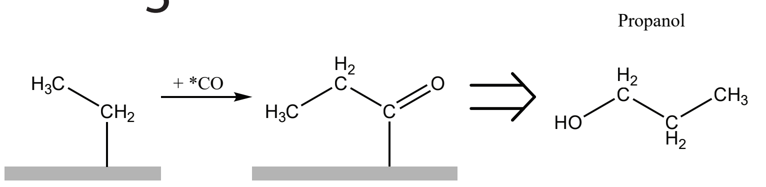

D-C_3

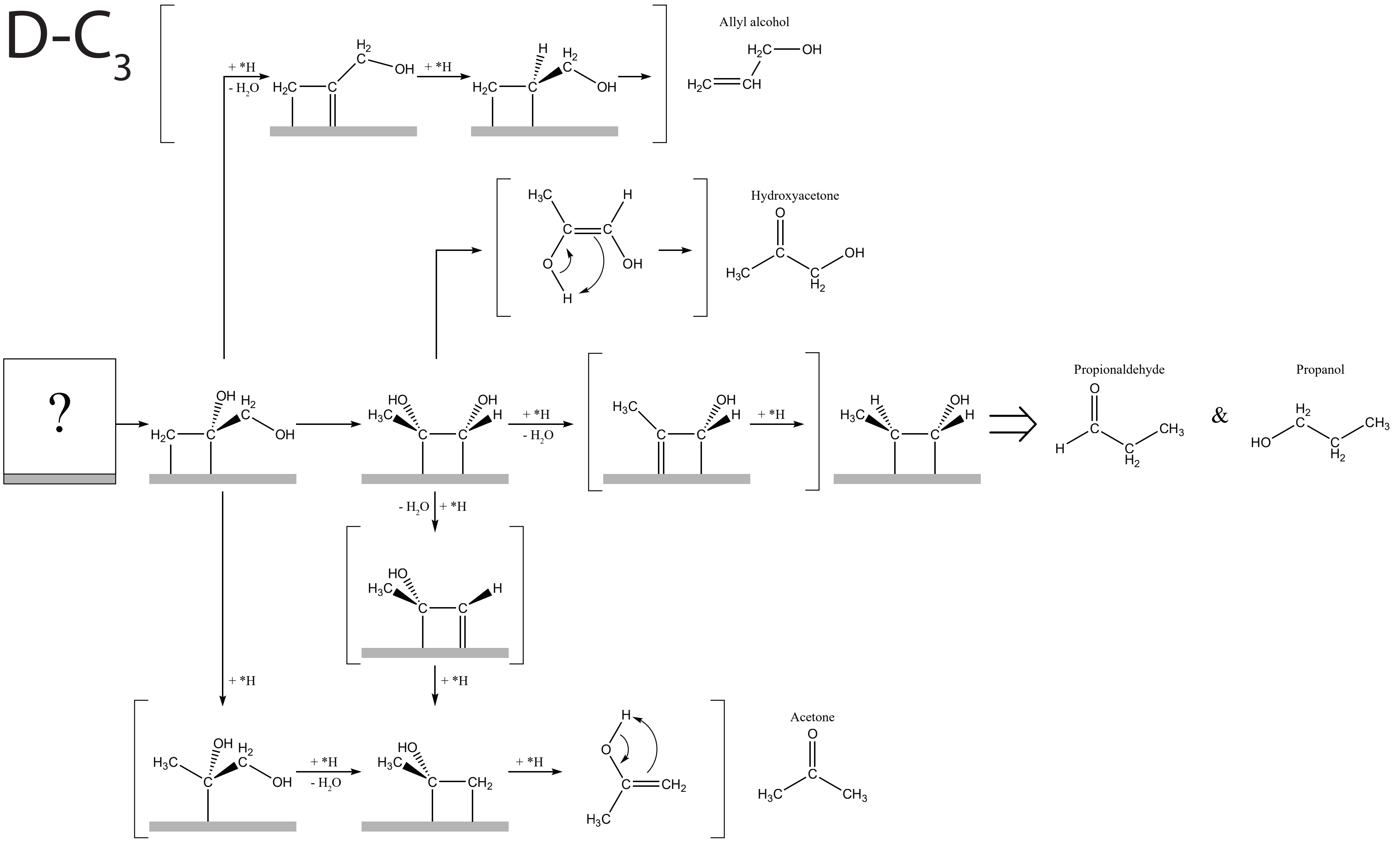

E-C_3

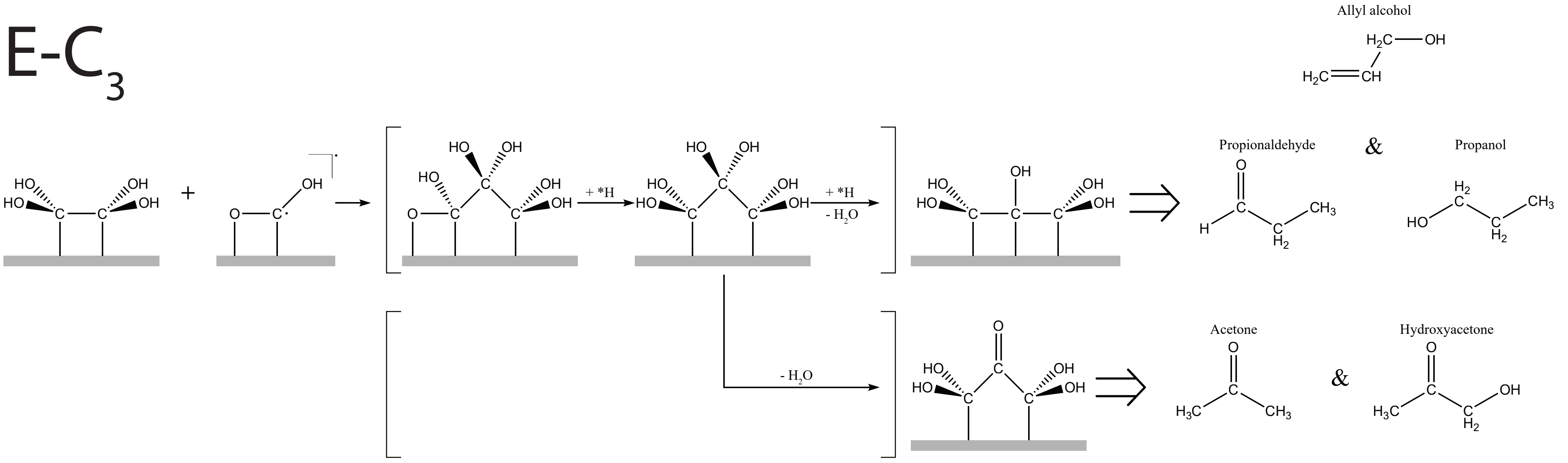

J-C_3

(Does not have a proposed C_2 mechanism, though they cite to match with A-C_2)

Ren, D.; Wong, N. T.; Handoko, A. D.; Huang, Y.; Yeo, B. S., Mechanistic Insights into the Enhanced Activity and Stability of Agglomerated Cu Nanocrystals for the Electrochemical Reduction of Carbon Dioxide to n-Propanol. J Phys Chem Lett 2016, 7 (1), 20-24.

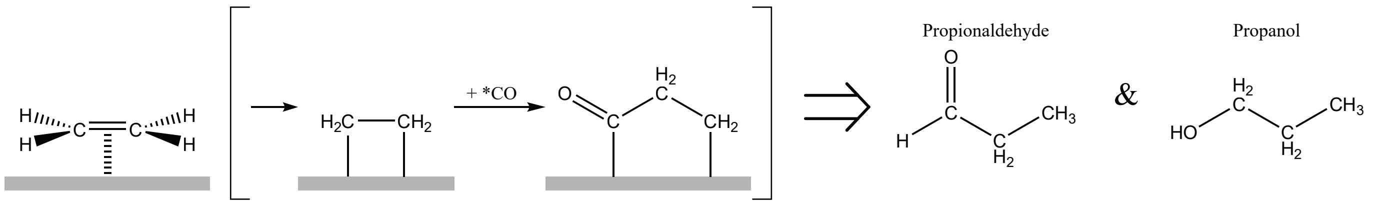

Supplement: Supplementary file 1 — Supporting Information [file ANIE-60-21732-s001.pdf]
